# A Novel Contactless Scanning Conductivity-Detection Approach for Moving Reaction Boundary Analysis in Electrophoresis Titration Sensors

**DOI:** 10.3390/s26072261

**Published:** 2026-04-06

**Authors:** Haozheng Dai, Youli Tian, Ke-Er Chen, Weiwen Liu, Qiang Zhang, Chengxi Cao

**Affiliations:** 1 School of Automation and Intelligent Sensing, Shanghai Jiao Tong University, Shanghai 200240, China; haozheng_dai@sjtu.edu.cn (H.D.); sjtu0579220@sjtu.edu.cn (K.-E.C.); weiwenliu@sjtu.edu.cn (W.L.); 2 School of Artificial Intelligence in Traditional Chinese Medicine, Shanghai University of Traditional Chinese Medicine, Shanghai 201203, China; tyl_sjtu@163.com; 3 State Key Laboratory of Microbial Metabolism, School of Life Sciences and Biotechnology, Shanghai Jiao Tong University, Shanghai 200240, China

**Keywords:** scanning contactless conductivity detection, moving reaction boundary, electrophoresis titration

## Abstract

Electrophoresis titration sensors are widely used for biomarker detection. However, traditional methods rely on a visible boundary for signal readout. Although conventional capacitively coupled contactless conductivity detection avoids indicator dependence, its single-point detection method suffers from long measurement times, large amounts of redundant data, and the inability to dynamically monitor the moving reaction boundary. To address these issues, we developed a novel contactless scanning capacitively coupled conductivity-detection method for microchip electrophoretic titration sensors. This method enables the rapid tracking and monitoring of the boundary within the microfluidic channel through dynamic scanning. The spatial distribution of conductivity during electrophoretic titration was theoretically analyzed. To evaluate the method, glucose was chosen as a model analyte. Quantitative detection was achieved over the linear range of 0.2–50 mM, with the limit of detection of 0.1 mM. The method exhibited satisfactory stability with relative standard deviation values ranging from 0.9% to 4.3% (*n* = 3). While the detection limit is higher than optical methods (0.02 mM), the results confirmed that the novel method offers merits, such as compact size, low cost and label-free operation. Moreover, it demonstrated strong potential for portable, quantitative analysis of target analytes across a wide range of applications.

## 1. Introduction

Electrophoresis titration (ET) sensors have shown significant application potential for the quantitative detection of biomarkers, including proteins [1], metabolites [2], and nucleic acids [3]. In ET sensors, ions from anolyte and catholyte reservoirs migrate in opposite directions under an electric field, reacting in the microchannel to form a reaction boundary. The boundary migrates progressively with the advancement of the reaction, which is called moving reaction boundary (MRB) [4]. The moving velocity of MRB usually correlates with the concentration of the target analyte. ET sensors have several advantages, including better selectivity, lower cost, enhanced portability, and simpler operation.

Traditional ET sensors, similar to thermometers, rely on a visible MRB and require chromogenic reagents (e.g., phenolphthalein, etc.), fluorescent labels, or colored ions, limiting their development and application. Furthermore, the dependence on visualization requires optical detection. It is primarily categorized into two approaches: naked-eye reading based on scale ruler and image detection using optical equipment. However, manual readings are subjective and prone to errors, while optical-instrument-based methods require bulky, expensive equipment (e.g., CCDs or fluorescence microscopes). Thus, the development of non-optical detection techniques for dynamic monitoring of MRB has grown in importance.

To address the above limitations, the capacitively coupled contactless conductivity detection (C^4^D) approach has emerged as a promising alternative technique [5,6]. Owing to its inherent advantages including simple structure and cost-effectiveness, C^4^D has become a universal detection technology widely used in the field of analytical chemistry [7,8]. However, conventional single-point C^4^D has several limitations for MRB detection [9]. First, its fixed-point nature prevents dynamic monitoring spatial position changes of the boundary and migration velocity of it. Second, the detector is typically placed near the end of channel, requiring the boundary to fully pass through to generate a complete signal peak, which increases detection time. Third, to avoid signal omission, the single-point detector must perform continuous conductivity monitoring. A lot of redundant data was collected and stored during monitoring, putting a strain on system resources and slowing down data processing and wireless transmission. Fourth, the single-point detection mode has low local interference immunity, which can lead to signal distortion and thus affect the detection accuracy. Therefore, addressing these single-point limitations is essential for the development of ET sensing.

An effective and innovative approach to avoid these issues is the emergence of scanning C^4^D (sC^4^D) [10]. By enabling electrodes to move freely with precisely controlled parameters (direction, position and velocity), sC^4^D facilitates whole-column detection [11,12]. Its flexibility is ideal for ET sensor integration. Based on this, our group previously developed the first automated sC^4^D system, which eliminated optical requirements and mobilization steps in isoelectric focusing (IEF), reducing detection time by ~96% while enhancing resolution [13]. Building on this established system, we herein firstly demonstrate the non-optical dynamic monitoring of MRB using sC^4^D.

Compared with conventional single-point C^4^D sensors, the proposed scanning approach enables spatiotemporal analysis for ET, overcoming the inherent limitations of fixed-point detection, including the inability to track boundary movement, prolonged detection time, and excessive redundant data. Compared with our previous sC^4^D system developed for IEF, the current MRB–sC^4^D platform incorporates three key advances. First, the microchip and PCB electrodes were redesigned to ensure reliable conductivity detection of MRB. Second, a dedicated signal processing strategy was developed to accurately identify the boundary position from scanned conductivity signals and to enable quantitative analysis based on time-resolved data. Third, sC^4^D is, for the first time, applied to ET sensors, demonstrating its feasibility as a quantitative analytical technique.

Herein, we first redesigned and fabricated the MRB microchip and PCB electrodes. Second, we used numerical simulation to characterize the conductivity distribution during ET. We then systematically optimized the excitation voltage and frequency of the C^4^D detector. Furthermore, we performed optical–electrical cross-validation to verify that electrical scanning can accurately detect the real boundary. Finally, using glucose as model biomarker to validate the feasibility of the analytical methodology, the quantitative standard curve was established, demonstrating the feasibility of this method as a basic analytical technique.

## 2. Materials and Methods

### 2.1. Chemicals and Regents

Leucocrystal violet (LCV), crystal violet, potassium chloride (KCl), horseradish peroxidase (HRP), glucose oxidase (GOD) and methanol were purchased from Shanghai Macklin Biochemical Industries, Shanghai, China. Sodium acetate and glucose (Glu) were obtained from Titan Scientific Co., Ltd. (Shanghai, China). Agarose with low gelling temperature was purchased from Sigma-Aldrich (Shanghai, China). All chemicals in this work were analytical-grade, and the preparation of solutions was carried out using Milli-Q water (Merck Millipore, Burlington, MA, USA).

### 2.2. Solutions

Stock solutions used in the work included 10 mM KCl, 10 mM sodium acetate, 50 mM glucose, 2 mM LCV in methanol, 10 mM crystal violet, 5 mg/mL HRP, and 10 mg/mL GOD. HRP and GOD were stored at −20 °C, with avoidance of repeated freeze–thaw cycles during use. The remaining stock solutions were stored at 4 °C. All reagents should be used within one week.

The working solutions were prepared with stock solutions. The crystal violet stock solution was serially diluted to prepare solutions with concentrations of 50, 100, 200, 500, and 1000 μM, which were used for experimental validation. The cathodic solution consists of 1 μM KCl and 2 μM sodium acetate. The gel buffer in the separation channel is 1% (*w*/*v*) low-temperature agarose gel that also contained 1 μM KCl and 2 μM sodium acetate. The anodic solution for the enzymatic reaction was prepared by mixing 1 mg/mL GOD (10 μL), 2 mM LCV (500 μL), 500 μg/mL HRP (10 μL), and 480 μL of the test solution.

### 2.3. Apparatus

The conductivity signals were obtained via an automated sC^4^D platform developed in our previous work [13]. The platform included a self-developed detection and drive module and a scanning device. The module was used for voltage excitation, signal conditioning and acquisition. Also, the module was responsible for driving the scanning device. The device used a single trackball screw sliding table to sweep the C^4^D electrode across the channel of the chip to achieve scanning detection.

### 2.4. Fabrication of the MRB Chip and PCB-C^4^D Electrode

The polymethylmethacrylate (PMMA) chip, as illustrated in Figure S1A, with dimensions of 80 mm × 20 mm × 4.6 mm, is equipped with four 24 mm-long separation channels. Each channel is furnished with two reservoirs at either end, each measuring 4 mm in diameter and 3.5 mm in depth. All channels share uniform dimensions of 200 µm in width and 100 µm in depth.

As shown in Figure S1B, the fabrication process of the MRB chip consisted of four primary steps: (i) First, a three-dimensional model of the chip was designed using software to define the geometry of the microchannels and reservoirs. (ii) The design was then converted into a G-Code program to generate the toolpaths required for machining. (iii) The chip structure was fabricated on a PMMA substrate via CNC milling. Following the machining process, it was securely bonded to a layer of 50 μm thick Teflon tape using adhesive bonding to seal the microchannels. The film is smooth and flat, with excellent electrical insulation and abrasion resistance. (iv) The final fabricated PMMA chip was ready for use. The chip fabrication and channel configuration remain unchanged for different analytes. The device serves as a versatile platform, with specificity determined solely by the biochemical reagents used. To detect different analytes, users only need to replace the specific recognition agents (e.g., the relevant enzyme) in the reaction buffer and the corresponding buffer electrolytes.

Prior to scanning, the chip was immersed in a 1 M NaOH solution and cleaned for 10 min using an ultrasonic cleaner (KQ-250E, Kunshan Ultrasonic Instrument Co., Ltd., Kunshan, China). It was then rinsed repeatedly with Milli-Q water and finally dried in a drying oven (XMTA-500T, Shanghai Shenguang Instrument Co., Ltd., Shanghai, China). The purpose of this pre-treatment is to detach and remove machining residues (debris) and micro-burrs adhering to the channel walls by the combination of the alkaline solution and ultrasonic cavitation, ensuring channel patency. In this work, a total of 20 chips were fabricated under identical conditions. The randomized chip rotation scheme for experiments was adopted: before each measurement, a chip was randomly selected from the 20 chips, after each use, the chip was cleaned and returned to the pool for subsequent random selection. The cumulative use of each chip was limited to no more than 5 times to ensure consistent surface properties.

The designed PCB-C^4^D electrode comprises two pairs of C^4^D electrodes, as shown in Figure S1C. Each pair has identical dimensions. The width of the actuator electrode and the pick-up electrode is 2.5 mm. The cross-length between them is 1.1 mm, and the gap size is 1.5 mm. The center-to-center distance between the two electrode pairs is 8 mm, ensuring each pair is positioned directly beneath a separation channel. After a sinusoidal excitation voltage was applied to the excitation electrode EX, the two associated Ex electrodes of channels ch1 and ch2 simultaneously applied this voltage to the two channels of the chip. The two detection electrodes, PE1 and PE2, then collected the detection signals of their respective channels, thereby enabling contactless conductivity detection. To enhance sensitivity and reduce noise interference from direct capacitive coupling between electrodes, the two sensing electrodes of each pair were placed in an antiparallel orientation [14]. All traces except the detection window were routed on the bottom layer of the PCB. Additionally, to provide electrical shielding and minimize noise, the entire remaining surface area is filled with a grounded copper pour, excluding the sensing electrodes, contact pads and signal traces [15].

### 2.5. Working Principle

**Figure 1:**
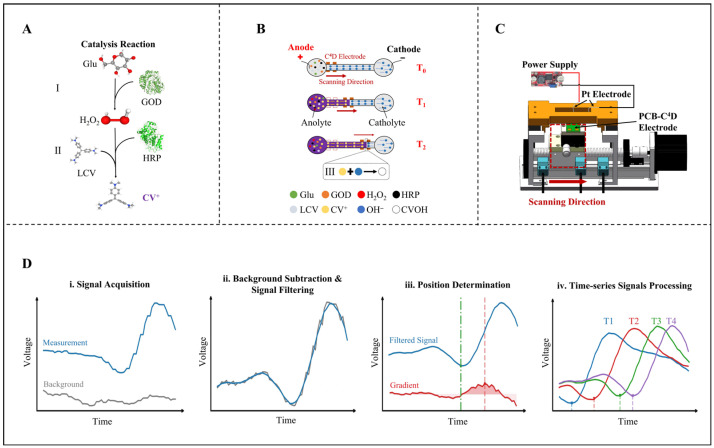
Schematic illustration of MRB–ET model, the sC^4^Dplatform and signal processing workflow. (**A**) Principle of CV^+^ production in the anode reservoir via catalytic reactions. (**B**) The mechanism of the MRB formed in agarose gel-filled channel. (**C**) Overview of the platform setup. (**D**) The workflow of the data-processing algorithm.

To verify the reasonableness and accuracy of signals measured by the proposed method, visualized signals are required as a reference. Thus, all ET experiments in the work were performed based on the LCV-based MRB–ET model previously proposed by our group [2]. The principle of the MRB–ET model was illustrated in Figure 1. In the anode reservoir, Glu was converted to the colored cation CV^+^ by a two-step enzyme-catalyzed reaction (Figure 1A). First, GOD oxidized glucose to generate H_2_O_2_. Second, under the catalysis of HRP, H_2_O_2_ further converted LCV to CV^+^:

(1) $$\mathrm{Glu}\;+\;O_{2}\text{-}\mathrm{GOD}\;\to \;\mathrm{gluconic}\;\mathrm{acid}\;+\;H_{2}O_{2}$$

(2) $$H_{2}O_{2}+\;2\mathrm{LCV}\overset{\mathrm{HRP}}{\to }{2H}_{2}O_{2}+\;2CV^{+}$$

Under the electric field, CV^+^ migrated into the reaction channel from the anode to the cathode. As the channel was pre-filled with agarose gel containing alkaline sodium acetate, CV^+^ reacted with OH^−^ to form colorless CV-OH. This titration reaction created a colored phase region and a colorless phase region in the channel, thereby generating a visible MRB, as shown in Figure 1B:

(3) $$CV^{+}\;+\;OH^{-}\;\to \;\mathrm{CV}\text{-}\mathrm{OH}$$

Figure 1C presents the diagram of this sC^4^D platform’s setup. The power supply connected to the Pt electrode to apply voltage for electrophoresis. During detection, this unit enclosed in the red dashed box, together with the PCB-C^4^D electrodes it carries, scans from the anode to the cathode following the red arrow, with the chip kept fixed.

To achieve quantitative analysis of the analyte concentration based on the collected conductivity signals, the data-processing algorithm was developed as illustrated in Figure 1D. Before electrophoresis, to establish a baseline, an initial scan of the pre-filled chip is recorded to account for any background variations. After scanning detection, the smooth signal is obtained by subtracting this baseline value from the measured data and applying a 5-point moving average filter. A gradient algorithm is then employed to precisely locate the moving boundary. The time derivative of the filtered sample signal is calculated. The starting point of the non-negative interval containing the maximum derivative peak is taken as the time when the boundary is detected. Combined with the scanning speed, the migration distance of the boundary can be determined. This algorithm enables precise boundary localization for multiple time-series signals. After finishing all data acquisition and signal processing, the distance was plotted against time to determine the average velocity (V_MRB_) from the slope. Since V_MRB_ correlates linearly with the logarithm of the substrate concentration according to the previous work [2], it serves as the quantitative basis.

### 2.6. Procedure of MRB–sC^4^D

First, a 1% (*w*/*v*) agarose gel solution was heated appropriately in boiling water until fully dissolved. The gel solution was then injected into the chip channels using a micro injector. Any excess gel in the anode and cathode reservoirs was removed once the gel had solidified.

Second, following gel loading, the chip was placed on the sC^4^D platform. The electromagnet was controlled to adsorb the cover plate embedded with a metal block so that it can press the chip downward tightly.

Next, the prepared glucose solution was mixed with solutions containing GOD, LCV, and HRP, and the mixture was incubated. After a 2 h catalytic reaction, the solution was added to the anode reservoir, while the cathode reservoir was simultaneously filled with cathodic electrolyte.

Then, the C^4^D detector was controlled by software to perform an independent scan from the anode to the cathode prior to the MRB–ET run, with the resulting signal recorded as the background signal.

Furthermore, an electric field of 30 V was applied between the cathode and anode to carry out MRB–ET on the chip.

Finally, upon completion of the experiment, all data were analyzed to extract the corresponding peak positions, allowing for final quantitative detection.

### 2.7. Optimization of C^4^D Excitation Signal Parameters

To evaluate the effect of amplitude of excitation signal, we tested five different excitation voltages (10, 15, 18, 22, and 25 Vpp) at an excitation frequency of 50 KHz. Experiments were performed under same conditions except for amplitude, using a 200 μM standard solution of crystal violet as the test sample.

Then, we tested five different frequencies, 35, 40, 50, 55, and 60 KHz, at an amplitude of 25 Vpp to determine the optimal frequency. The experiments were still performed using a 200 μM standard solution of crystal violet as the test sample.

### 2.8. Quantitative Detection of Glucose

To determine the dynamic range and limit of detection (LOD) of the platform, glucose solutions with different concentrations (0.2, 0.5, 1, 3.5, 10, 30, 50 mM) were prepared for the reaction. During the detection process, the boundary inherently moved simultaneously. According to our previous studies [13], when the scanning speed is less than or equal to the MRB migration speed, the detector can never catch up with the boundary, making it impossible to detect the signal peak. Conversely, if the scanning speed is too high, significant signal distortion may occur, interfering with accurate detection. Within an appropriate range, variations in scanning speed do not substantially affect the measurement of the velocity of MRB. Therefore, the selection of scanning speed represents a trade-off between reliable boundary tracking, signal fidelity, and analysis time. Based on this consideration, 1.0 mm/s was selected as an optimal compromise in this work. After the start of the reaction for 7 min, we started to collect conductivity data. This constant delay was adopted to guarantee sufficient formation of MRB and its entry into the scanning region. At the end of the unidirectional scan, the detector returned to the starting point and waited for the next scan to start. The interval between the two scans was 2 min, and the experiment was completed after 16 min. Peak detection was performed on the signal, and the data of the first four valid signal peaks acquired were taken for linear fitting to calculate the velocity of MRB. If there are fewer than four valid data points, use at least three data points for fitting.

## 3. Results

### 3.1. Confirmation of MRB–sC^4^D Signal

**Figure 2:**
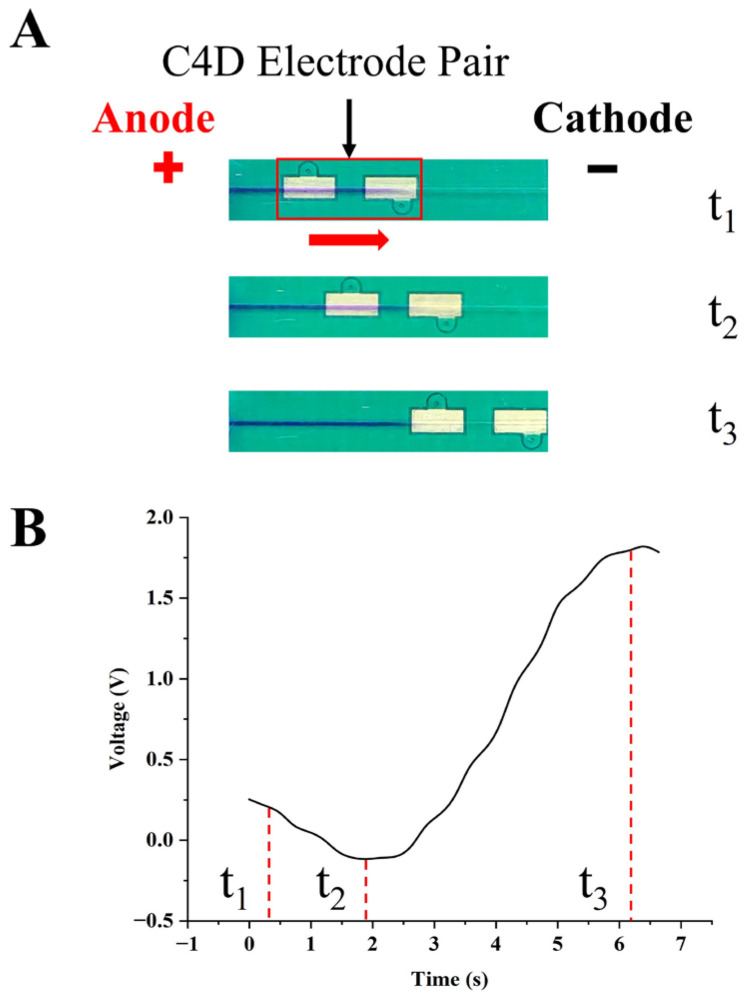
Confirmation of MRB–sC^4^D boundary detection. (**A**) Dynamic images captured using a camera of an electrode pair scanning through the boundary from t_1_ to t_3_; (**B**) Conductivity signals detected during scanning from t_1_ to t_3_.

To make sure that the detector scanning past the boundary did detect the corresponding signal peaks, we simultaneously imaged the scanning process using a camera. In Figure 2, the distinct boundary was formed within the channel. The C^4^D electrode pairs, labeled by solid red box, moved from the anode to the cathode. We recorded the entire scanning process of the electrode pair, starting from its initial position (when the boundary was about to enter the sensing region) until it completely scanned across the boundary. Figure 2A presented the images taken at three major time points during this scanning process, labeled sequentially as t_1_ to t_3_. Figure 2B showed the conductivity signals recorded synchronously throughout the process. As the electrode pair traversed the boundary, a peak of the negative conductivity signal gradually emerged. Comparative analysis of the images and conductivity signals at each time point illustrated that the conductivity signal initially decreased as the electrode pair just passed through the boundary and peaked when the pick-up electrode has completely left the boundary. Subsequently, the conductivity signal gradually increased as more of the sensing region passed through the boundary. The formation mechanism and theoretical analysis of the signal have been elaborated and explained in detail in Section 3.2.

The results presented in Figure 2 demonstrated that the sC^4^D enabled valid signal detection of the boundary. Moreover, when the scanning speed of the sensor was held constant, the instantaneous physical position of the boundary could be further calculated using the values of t_1_, t_2_ and t_3_ corresponding to the signal peak. For the subsequent analyses in this work, all boundary positions were derived from calculations based on the value of t_2_.

### 3.2. Theory of the MRB–sC^4^D Model and Numerical Simulations

**Figure 3:**
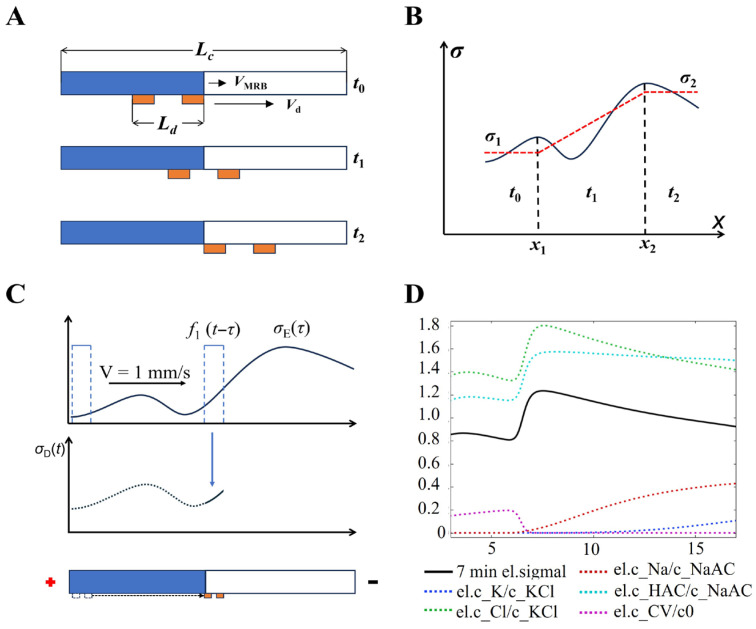
Schematic illustration of the MRB–sC^4^D model. (**A**) Schematic of MRB detection using sC^4^D. (**B**) Schematic illustration of the conductivity distribution along the channel. (**C**) Schematic diagram of conductivity detection by detector using a sliding-window function. (**D**) Ion concentration and conductivity distribution within the electrophoresis channel at 7 min in the numerical simulation model.

Conventional microchannel conductivity detection typically operated in a single-point mode, monitoring the temporal changes of ion migration within a localized sensing region. In this work, by employing scanning detection, we achieved monitoring of the spatial conductivity distribution throughout the entire channel. Figure 3 showed the boundary at a representative time under idealized conditions. Since the scanning speed of the detector was much higher than the migration velocity of the MRB, the boundary was regarded as stationary during the measurement. The scanning process across the boundary can be divided into three stages: before reaching the boundary (t_0_), while traversing the boundary (t_1_), and after completely passing through it (t_2_).

According to previous work, the conductivity signal within the channel during electrophoresis can be described by the following equation [16]:

(4) $$\sigma =F\left(\mu_{eff}^{+}c^{+}+\mu_{eff}^{-}c^{-}\right)$$

The MRB generated by ET was assumed to form an ideal sharp boundary moving from left to right. The conductivity on the left side of the boundary was denoted as $\sigma_{1}$, while that on the right side was $\sigma_{2}$. Within the boundary region, the conductivity was assumed to vary linearly from *σ*_1_ to *σ*_2_ (assuming that $\sigma_{1}$ < $\sigma_{2}$), as illustrated by the red line in Figure 3B. Under these conditions, the conductivity can be expressed as:

(5) $$\sigma =\frac{\sigma_{2}-\sigma_{1}}{x_{2}-x_{1}}x$$

The electrophoresis channel was filled with agarose gel to suppress fluid convection, while ions migrated from the electrode reservoirs into the channel. At the solution–gel interface, differences in diffusion and flux among various species, together with ion migration during electrophoresis, led to a nonlinear distribution of conductivity signals within the MRB region, as illustrated by the black solid line in Figure 3B. During the titration process, a region of low conductivity is formed at the leading edge of the moving boundary, driven by two key factors: the inconsistent migration velocities of the ions involved and the gradual depletion of OH^−^ ions.

When a low-conductivity region is present in the ion migration path, the ion migration velocity within this region is significantly reduced. This reduction slows the entry of anions into the region, leading to anion aggregation and the formation of localized high-conductivity zones. The accumulated anions further increase the anion concentration in these localized regions; given the high ionic mobility of anions, the concentrated buildup results in a localized elevation of conductivity in the region.

Therefore, MRB detection can be performed by scanning the conductivity signal within the channel. In this case, the output of detector can be regarded as the signal of the electrophoresis channel after processing with a sliding-window function during scanning (as shown in Figure 3C). At a given electrophoresis time *τ*, the signal obtained by scanning can be expressed as:

(6) $$\sigma_{D}\left(t\right)=\sigma_{E}\left(\tau \right)\cdot f\left(t-\tau \right)$$

In the above expression, $\sigma_{E}\left(\tau \right)$ represents the conductivity distribution within the channel at the given time *τ*, f denotes the scanning function determined by the detector, and $\sigma_{D}\left(t\right)$ corresponds to the conductivity output recorded by sC^4^D.

We performed numerical simulations on the MRB–sC^4^D model. The results indicated that, upon MRB formation, both the types and concentrations of ions differ across the boundary (as illustrated in Figure 3D). The conductivity signal at the boundary typically exhibited a step-like profile. Moreover, the signal along the channel reflected the spatial distribution of the various ions throughout the system.

In the simulation, to simplify the analysis, it was assumed that the boundary movement remained stable, and the effects of other ion migrations were neglected. Under this assumption, the concentration distribution of CV^+^ within the channel was described by the following equation [17,18,19]:

(7) $$c_{CV}(x,t)=c_{0}(1-erf(\frac{x-Ut}{2\sqrt{Dt}}))$$

here, *x* denotes the spatial position, *c*_0_ is the characteristic initial concentration, *U* represents the migration velocity, and *D* is the diffusion coefficient.

Similarly, the conductivity signal resulting from the ion distribution assumed in Figure 3B can be expressed as:

(8) $$\sigma_{E}\left(x,t\right)=\sigma_{1}+\frac{\left(\sigma_{2}-\sigma_{1}\right)}{2}erf(\frac{x-Ut}{2\sqrt{Dt}})$$

According to Equation (3), the signal recorded by sC^4^D at a given electrophoresis time *τ* can be expressed as:

(9) $$\sigma_{D}\left(t\right)=\sigma_{E}\left(x,\tau \right)\cdot f\left(t-\tau \right)$$

here, the function *f* represents the sliding-window function determined by the detector.

During actual ET process, the solution–gel interface at the channel inlet exhibits a steep concentration gradient and significant spatial variation. Moreover, differences in the diffusion coefficients and electrophoretic mobilities of various ions further contribute to deviations of the MRB interface from the ideal state, resulting in fluctuations.

Overall, the above theory and numerical simulations provide a basis for understanding the signal formation mechanism and detection principle of the MRB–sC^4^D model (for more details, see Figures S2–S4 in Section S2 of the Supplemental Materials). The diffusion coefficients and electrophoretic mobilities of ions in the numerical simulation model were listed in Table S1.

### 3.3. The Optimization Experiment of Excitation Signal

**Figure 4:**
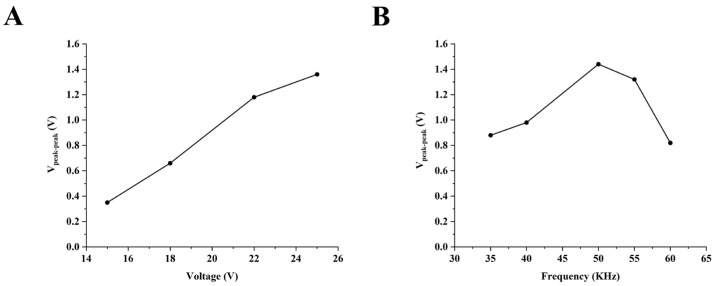
The results of sC^4^D detection response with different excitation voltages and excitation frequencies. (**A**) The peak-to-peak values of the detected signals at different excitation voltages (frequency: 50 KHz). (**B**) The peak-to-peak values of the detected signals at different excitation frequency (voltage: 25 Vpp).

We first tested five different excitation voltages (10, 15, 18, 22, and 25 Vpp) at an excitation frequency of 50 KHz. After calculating the peak-to-peak values of the detected signals obtained at different voltages, the results were shown in Figure 4A. When the excitation voltage was set to 10 Vpp, no distinct signal peaks were detected. The results indicate that the peak-to-peak values of the measured conductivity signals decreased as the excitation voltage amplitude was reduced. Therefore, to improve the detection signal-to-noise ratio, the final excitation voltage amplitude was set to 25 Vpp.

After determining the optimal excitation voltage, we proceeded to optimize the excitation frequency. As shown in Figure 4B, the output signal increased and then decreased with the rise of the excitation frequency, and the optimal excitation frequency was 50 KHz. This is because, when the excitation frequency is lower than the optimal value, the coupling capacitance in the C^4^D system exhibits excessively high capacitive reactance. As the excitation frequency increases, this capacitive reactance decreases progressively, which in turn leads to an increase in the output voltage. In contrast, when the frequency exceeds the optimal value, the dominant influence arises from the capacitance of parasitic components—such as the bypass capacitance formed by direct coupling between the electrodes [20]. Given that these parasitic capacitors act as parallel branches in the circuit, the reduction in their capacitive reactance (with increasing frequency) causes more significant current shunting [21]. This shunting effect ultimately results in a decrease in the final output signal.

In summary, the optimized excitation parameters were determined to be 25 Vpp at 50 kHz.

### 3.4. Demonstration of MRB–sC^4^D for ET Sensing

**Figure 5:**
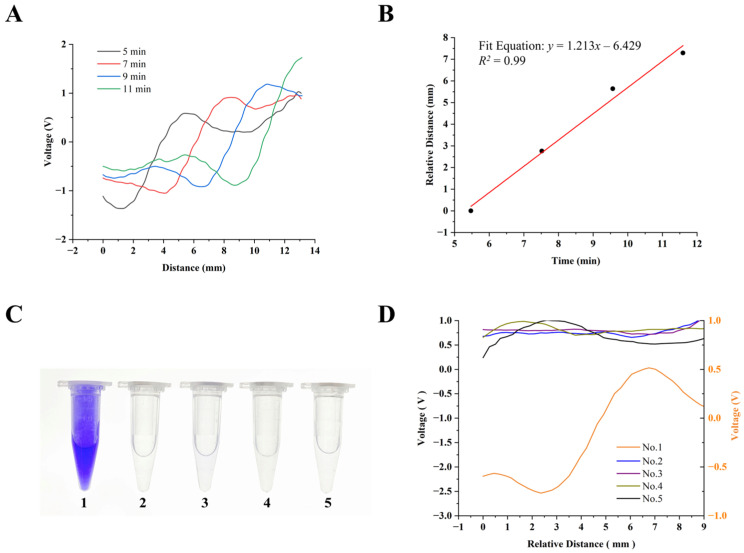
Experimental verification of the MRB–sC^4^D approach for ET-sensing. (**A**) Summary plots of conductivity signals from 4 scans performed at 2 min intervals over a period of 5–11 min. (**B**) The linear fitting curve between the relative distance of boundary movement and the running time of (**A**). (**C**) Plots of Glu–GOD catalyzed reactions: (1) 1 mM Glu, 1 mg/mL GOD, 2 mM LCV and 0.5 mg/mL HRP; (2) 2 mM LCV and 0.5 mg/mL HRP; (3) 1 mg/mL GOD, 2 mM LCV and 0.5 mg/mL HRP; (4) 1 mM Glu, 2 mM LCV and 0.5 mg/mL HRP; (5) 1 mM Glu, 1 mg/mL GOD and 2 mM LCV. (**D**) Corresponding plots of scanning conductivity signals of Glu–GOD catalyzed reactions of (**C**).

Since MRB–ET in this work primarily relies on the reaction between CV^+^ and OH^−^, we first used the crystal violet standard solution for initial experiments to verify the feasibility of sC^4^D in MRB–ET sensing. When an electric field was applied, CV^+^ in the anode migrated well into the channel and reacted with the alkaline buffer of sodium acetate, forming a clearly visualized boundary. As the ET process proceeded, this boundary moved uniformly toward the cathode. Following electrophoresis initiation, the scanning and detection of C^4^D were performed at 2 min intervals, and the corresponding data were recorded. The signals from each of the four scans were shown in Figure 5A. The results clearly indicated that the acquired signal peaks displaced significantly as the reaction progressed. After calculating the trough position of each signal, the relationship between the relative boundary movement distance and ET running time was displayed in Figure 5B. It showed a good linear regression. Apparently, this experiment effectively demonstrated that sC^4^D was capable of monitoring the boundary movement distance. In addition, as shown in the overlaid conductivity profile in Figure S5, the experimental results were in great agreement with the numerical simulation results presented earlier. The signal peaks exhibited similar shapes, and the overall trends of the simulated curves were largely consistent, further verifying the accuracy of the theoretical and numerical simulation models.

Furthermore, validated enzymatic catalytic reactions were performed to verify that sC^4^D could be used for the ET model of Glu. Figure 5C showed the results of the catalytic reactions carried out in the presence of some or all of the components of Glu, GOD, LCV, and HRP. In terms of visual results, purple CV^+^ was only observed in Figure 5C (1) (the complete reaction system containing Glu, GOD, LCV and HRP). No similar color reaction occurred under other conditions. Meanwhile, as illustrated in Figure 5D, after the same ET running time, only the solution that had color reaction generated valid signal peak, while the other groups showed no obvious signals. Overall, as the results in Table 1, each component added to the catalytic solution contributed to the formation of the CV^+^ in the system. These results confirm that the reagents themselves do not contribute to background interference. Additionally, the conductivity signal detected must result from the reaction following migration of CV^+^. This confirms that sC^4^D can be applied to the ET model for Glu detection and analysis.

### 3.5. Quantitative Analysis of Glu

**Figure 6:**
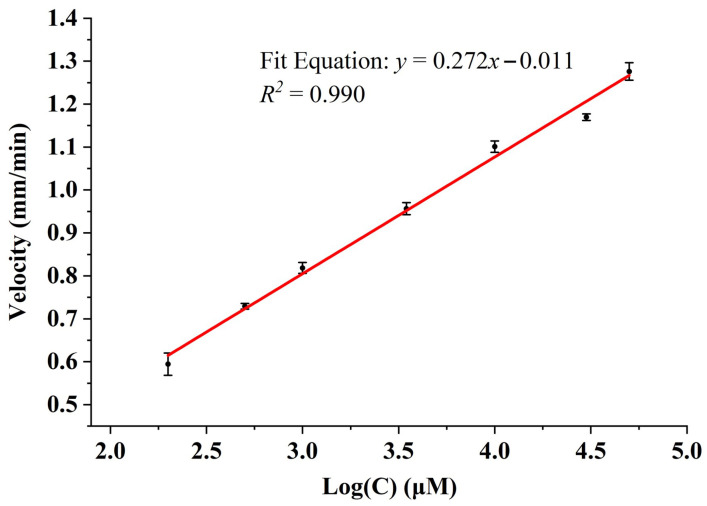
The linear fitting curve between the velocity of boundary movement and the logarithm of Glu concentration.

To quantitatively evaluate the analytical performance of the developed method, the relationship between the concentration of Glu and the boundary migration velocity was systematically investigated. As illustrated in Figure 6, there was a good linear relationship between the moving velocity of the boundary and logarithm of Glu concentration within a linear range of 0.2–50 mM. The corresponding linear regression equation was: *y* = 0.272*x* − 0.011, accompanied by a satisfactory correlation coefficient (R^2^ = 0.990). Notably, this broad linear range comfortably satisfied clinical requirements and demonstrated potential for clinical diagnosis [22]. Furthermore, the LOD of the proposed method was as low as 0.1 mM, indicating sufficient sensitivity for quantification. To assess the repeatability of the established method, three replicate experiments were performed for each concentration to measure its relative standard deviation (RSD). The low RSD values were 4.34%, 0.90%, 1.55%, 1.47%, 1.22%, 0.66% and 1.61%, suggesting fair stability. In addition, because chips were randomly selected for each measurement, the low RSD value demonstrates that chip-to-chip variability does not significantly affect analytical performance.

It is worth noting that the reported dynamic range can be further expanded through specific system adjustments. For higher analyte levels, the detection limit can be extended by diluting the sample to the linear range first. Alternatively, shortening the scanning interval to capture multiple boundary positions to calculate the moving velocity. Additionally, increasing the C^4^D scanning speed and sampling frequency enables precise tracking of high-velocity moving boundary without signal distortion.

For lower concentrations, the boundary typically moves slower, and the conductivity signal strength is weaker. To address this, one approach is to extend the sampling interval, allowing the boundary to move a sufficient distance for detection. Another option is to use low-conductivity buffers in microfluidic channels to improve the signal-to-noise ratio and enhance detection sensitivity. Additionally, the design of C^4^D electrodes can be optimized to increase sensitivity [23]. Specifically, increasing the electrode width strengthens the signal but may cause peak broadening. Reducing the electrode spacing improves spatial resolution and sensitivity, but it also leads to a significant decrease in signal amplitude [24]. Therefore, the optimization process necessitates a careful balance based on the detection requirements. Also, we can attempt to use a thinner insulator layer with a high dielectric constant to enhance the capacitive coupling [25]. Finally, increasing the excitation voltage and optimizing the frequency can maximize sensitivity. Since the signal conditioning circuit is self-developed, further improvements of the circuit can be made to boost signal gain and reduce system noise.

Recovery experiments were performed by spiking the test samples with different concentrations of analyte standard solutions, aiming to evaluate the impact of matrix effects on analytical performance. To demonstrate the applicability of this method to alcoholic beverages, the standard solutions of glucose were spiked into sugar-free beer in this study. All samples were diluted 1:100 prior to analysis after spiking. The results of the recovery were 89.1%, 98.6% and 107.7%, respectively, with RSD all below 10% (Table 2). The results confirmed the reasonable accuracy of the method.

## 4. Discussion

The developed MRB–sC^4^D platform demonstrates the following merits over traditional approaches, offering an alternative for portable sensing. To begin with, we compared it with the single-point C^4^D method. In fact, the sC^4^D method has inherent methodological advantages, as demonstrated in our previous work on IEF [13]. These advantages exist in different electrophoresis modes (whether IEF or MRB). First, the efficiency of sC^4^D is definitely higher than that of single-point C^4^D. For sC^4^D, detection time is largely independent of sample concentration, with all samples detectable within the same set period. In this work, a single-channel whole-column scan takes under 30 s, and the total test time is under 16 min, which can be further shortened by optimizing the scan speed. In contrast, using single-point C^4^D results in varying detection times for samples of different concentrations. For example, the difference of MRB moving velocity between high-concentration (50 mM) and low-concentration glucose (0.2 mM) exceeds two-fold, causing a similar variation in detection time. In particular, for low-concentration glucose, it takes at least 10 min to pass through the detection window, and if the electrodes are positioned near the end of channel, the time from boundary formation to complete pass through the detection window could exceed 30 min. The detection time is extremely long. Second, since the boundary only passes through the detection window once, single-point C^4^D cannot dynamically monitor the ET process, and thus fails to capture kinetic information effectively. At last, sC^4^D offers greater flexibility and stability, enabling quick adjustment of scanning parameters (direction and speed) for different requirements. Conversely, fixed-position detectors require new sensor fabrication for position changes. This process is cumbersome, time-consuming, and costly.

In addition, we compared this method with traditional optical ET method. As we can see from Figure S6, the dynamic range of the optical ET was indeed wider (0.1–50 mM) and the limit of detection was 0.02 mM. We think it is due to the signal transduction mechanism. For low concentration, the colored boundary’s visual contrast is still high, but the conductivity change relative to the background buffer is small, leading to a lower signal-to-noise ratio.

However, because this method transforms the MRB detection mechanism from visualized real-time monitoring to dynamic conductivity analysis, we believe sC^4^D has several advantages in areas where optical methods are insufficient. First, it eliminates the need for light sources, expensive cameras, or fluorescence microscopes. So, this platform was low-cost, with the cost of the whole device and chip manufacturing not exceeding 100 dollars. And the absence of optical detection avoids complex image post-processing. It can monitor the ET process to obtain kinetic information, similar to optical detection, although with slightly lower real-time performance. Finally, the platform has the potential to detect colorless boundaries, overcoming the limitation of requiring colorimetric indicators or fluorescent markers. This method can further facilitate the development of MRB theory and provide broader detection potential for analytes that form colorless boundary. Therefore, in application scenarios where optical alignment is challenging, ambient light interference is present, or labeling procedures are restricted, the proposed MRB–sC^4^D method offers distinct advantages over conventional ET or microchip electrophoresis approaches. Taking creatinine, a typical physiological biomarker, as a representative example, conventional ET typically relies on chromogenic systems to convert creatinine into colored ions for boundary visualization, followed by optical imaging for detection. In contrast, our method eliminates the need for optical components, significantly reducing overall system complexity and cost. Similarly, in conventional microchip electrophoresis methods, creatinine detection mostly depends on ultraviolet (UV) absorbance [26,27], which is inherently tied to UV light sources and specific detectors. Our approach effectively circumvents this limitation. Based on our preliminary validation results, as shown in Figure S7A, the detectable concentration range of creatinine using the proposed method is 0.16–2.60 mM under current conditions.

Moreover, while glucose was employed as a proof-of-concept analyte in this study, the platform can serve as a universal detection solution, applicable to a wide range of clinically relevant biomolecules. It can be readily adapted to other targets simply by substituting glucose oxidase with other specific enzymes that catalyze the production of H_2_O_2_. For example, we achieved the quantitative detection of creatinine and choline by replacing the enzyme (as illustrated in Figure S7). The results confirmed that this platform can detect multiple physiological biomarkers. When the physiological or pathological state changes, the concentrations of related biomarkers are typically altered (e.g., diabetes results in elevated glucose levels; liver dysfunction leads to higher choline concentration and kidney failure causes a rise in creatinine concentration, etc.). This will affect the enzyme reaction products and eventually leads to differences in MRB migration velocity. By measuring the velocity with sC^4^D method, the platform can assess these physiological states, which will be a key focus of our future research.

Obviously, the method still has limitations at present as well. First, since glucose monitoring has been extensively studied and numerous mature products are already available, the detection performance of our method is relatively weak compared with existing electrochemical, optical glucose sensors or commercial instruments. As illustrated by the comparison results in Table 3, this method exhibits a relatively higher limit of detection than most electrochemical and optical glucose sensors, resulting in slightly lower sensitivity. But its linear range remains comparable to those of existing sensors and fully covers the clinically relevant glucose monitoring range, demonstrating strong potential for practical applications. It is worth noting that in this work, glucose is used only as a model analyte to benchmark the analytical performance of the proposed MRB–sC^4^D platform, rather than for the development of a dedicated glucose sensor. Certainly, optimizing and improving the glucose detection performance based on this method is also one of our research goals for the future.

Second, since the platform detects changes in conductivity, biological samples with high background ionic strength (such as undiluted serum) contain high concentrations of other salt ions, which can increase the background conductivity in the separation channel during electrophoresis, thus reducing the signal-to-noise ratio and even obscuring the signal peak. This necessitates further optimization of the components in the reaction reagent and concentrations of them, as well as improvements to the conductivity detection device to enhance its resolution.

Third, to focus on validating the MRB–sC^4^D detection principle and the conceptual and functional feasibility of the method, a simplified chip design was employed in the work. The complex passive or active micromixer structures were not incorporated (e.g., serpentine channels) on the chip. Instead, off-chip vortex mixing and incubation in microtubes ensured homogeneous mixing and complete reaction, thereby guaranteeing the reliability of substrate concentrations for subsequent detection. However, the automation capability of the platform was limited by this approach. Therefore, we will focus on integrating on-chip mixing structures and reaction chambers to eliminate manual handling in the future, achieving a fully automated workflow and better meeting the needs of point-of-care testing (POCT) applications. Similarly, we did not systematically evaluate the long-term stability of the chips in this study. However, no obvious performance degradation was observed within the scope of our experiments. Future work will involve dedicated investigations into chip durability, cleaning protocols, and the maximum number of reuse cycles to better support practical applications.

Moreover, in this study, we are focusing on analytes that generate H_2_O_2_ through enzyme reactions. Analytes that do not produce H_2_O_2_ for subsequent colored ion formation (such as dehydrogenase substrate and hydrolase substrate) will require further research into alternative signal transduction strategies. However, it is important to clarify that the dependence on H_2_O_2_ generation in this study is due to the specific MRB chemical reaction model chosen (H_2_O_2_-HRP-LCV model), rather than an inherent limitation of the sC^4^D detection platform itself. This model was selected because the colored boundary generated by the colored ions allows for simultaneous visual confirmation and conductivity detection, making it suitable for the conceptual validation in this study. In fact, the basic principle of the proposed platform is to detect conductivity changes across the MRB boundary instead of the visual signals from the colored boundary. Therefore, theoretically, this work can further extend the applicability of MRB in detecting physiological biomarkers. It provides broader detection potential for analytes and reaction systems that form colorless moving boundary, which are difficult or impossible to color. We will focus on validating the platform’s performance in these non-colored MRB models and expanding its application to additional MRB models in future work.

The current data processing approach depends heavily on manual operations and is rather time-consuming. To better fit the requirements of POCT, we will further optimize the whole system toward miniaturization, portability, and automation. Currently, we are developing a smartphone-based sC^4^D platform. It will support wireless data transmission and integrate data analysis algorithms in the software for signal processing, which helps improve detection efficiency. Once the smartphone-based system is integrated, the same overlay analysis between simulation and experimental results will be performed to validate the reliability of the developed portable system. In future work, the new platform will be further applied to practical research in areas such as biomedical diagnostics and food analysis.

## 5. Conclusions

In this work, we proposed a novel non-optical detection method (MRB–sC^4^D) for MRB–ET. Unlike traditional optical detection methods that rely on visible boundary, this non-optical approach offers advantages such as low cost, simple apparatus, and broad application potential. To establish the theoretical foundation of this method, we thoroughly elucidate the formation mechanism of the conductivity signal in the MRB–sC^4^D process. In addition, we performed systematic MRB–sC^4^D experiments for the detection of glucose. First, the key parameters of the C^4^D system were optimized. The method was further verified by cross-validation using optical detection. Finally, quantitative analysis was performed for glucose standards over a wide concentration range. The results confirmed the feasibility of the proposed method for the quantitative analysis of small molecules and it has good application prospects in the field of food processing or health monitoring in the future.

## Figures and Tables

**Table 1:** Glu–GOD catalyzed reactions under various conditions.

No	Glu	GOD	LCV	HRP	Color	sC^4^D
1	√	√	√	√	+	+
2			√	√	-	-
3		√	√	√	-	-
4	√		√	√	-	-
5	√	√	√		-	-

Note: ‘√’ means the reagent was added to the catalyzed reactions. ‘+’ means significant visual or conductivity signal; ‘-’ means no signal.

**Table 2:** Recovery of MRB–sC^4^D for Glu detection in sugar-free beer (*n* = 3).

Samples	Spiked (mM)	Found (mM)	Recovery (%)	RSD (%)
1	100	89.1	89.1	8.6
2	150	147.8	98.6	5.4
3	350	376.8	107.7	2.6

**Table 3:** Comparison of detection performance between the MRB–sC^4^D and other glucose sensors.

Sensors Type	LOD (μM)	Linear Range (mM)	References
Electrochemical Sensor (Enzymatic)	4.6	0.2–31.6	[28]
	50	0.15–3	[29]
	198	1–16.5	[30]
Electrochemical Sensor (Non-Enzymatic)	3.1	0.01–21	[31]
	26.17	0–15	[32]
	268	1–30	[33]
Optical Sensor	1	2–14	[34]
	10	0.1–20	[35]
	193	0–24	[36]
Commercial Glucometer (Dexcom G6)	~0.5 mM	2–22	[37]
Commercial Glucometer (Contour Next One)	~1.1 mM	1.1–33.3	
MRB–sC^4^D	0.1 mM	0.2–50	This work

## Data Availability

The original contributions presented in this study are included in the article/Supplementary Materials. Further inquiries can be directed to the corresponding authors.

## Supplementary Materials

The following supporting information can be downloaded at: https://www.mdpi.com/article/10.3390/s26072261/s1, Figure S1: Schematic diagram of the designed MRB chip and manufacturing process flowchart, along with the schematic diagram of the PCB-C^4^D electrode; Figure S2: Numerical simulation model of MRB–sC^4^D; Figure S3: Numerical simulation results of ion concentration and conductivity distributions in the electrophoresis channel at an electrophoresis time of 11 min; Figure S4: Simulation results of conductivity distributions in the channel at different time; Figure S5: Overlay of the simulated and experimental conductivity profiles. Figure S6: The experimental results of optical ET for Glu detection. Figure S7: The quantification detection of creatinine and choline. Table S1: Diffusion coefficients and electrophoretic mobilities of ions in the numerical simulation model.
