# Supplementary material for: A Novel Contactless Scanning Conductivity-Detection Approach for Moving Reaction Boundary Analysis in Electrophoresis Titration Sensors
Source: Sensors (Basel). 2026 Apr 6;26(7):2261. doi: 10.3390/s26072261 (PMC13075292; doi:10.3390/s26072261)
Supplement: Supplementary file 1 [file sensors-26-02261-s001.zip › sensors-4214590_supplementary.pdf]

Supplemental Material

A Novel Scanning Contactless Conductivity Detection Approach for Moving Reaction Boundary Analysis in Electrophoresis Titration Sensors

Haozheng Dai, Youli Tian, Ke-Er Chen, Weiwen Liu, Qiang Zhang, and Chengxi Cao

Abbreviations: ET, Electrophoresis Titration; **MRB**, moving reaction boundary; **sC⁴D**, scanning capacitively coupled contactless conductivity detection; **Glu**, glucose

S1. Fabrication of the MRB chip and PCB-C⁴D electrode

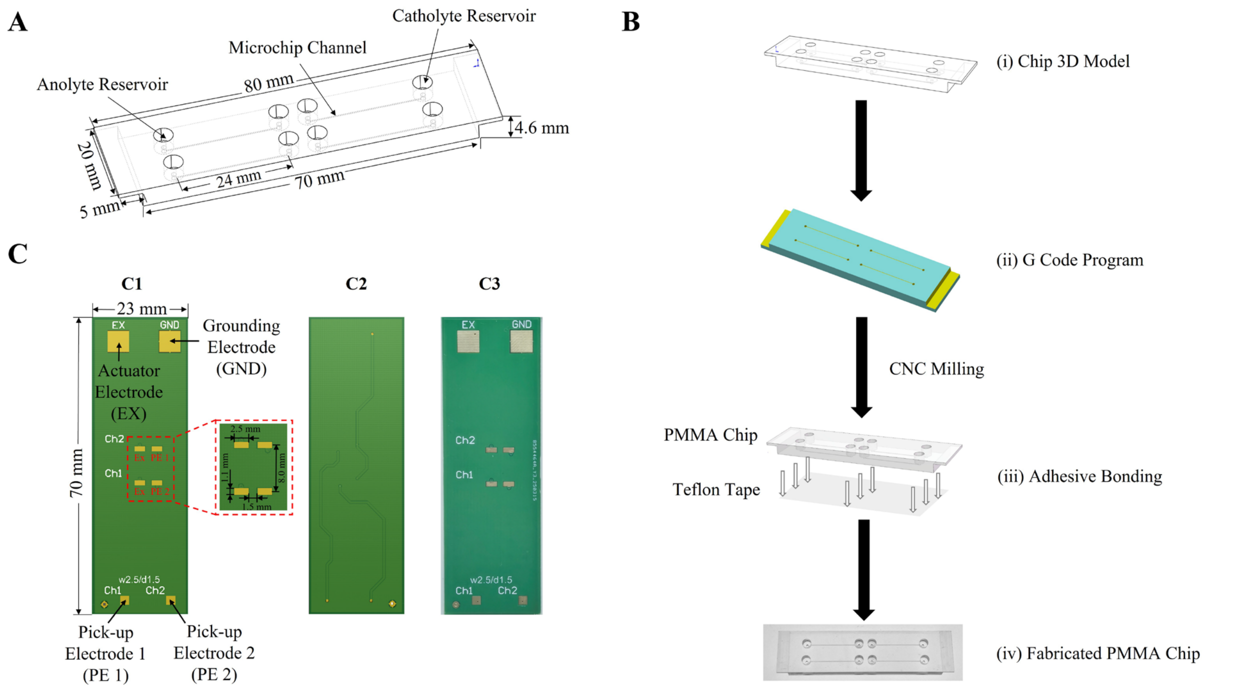

Figure S1. Schematic diagram of the designed MRB chip and manufacturing process flowchart, along with the schematic diagram of the PCB-C⁴D electrode. (A) Three-dimensional schematic of the MRB chip. (B) Manufacturing process. (C) Dimensions of the PCB-C⁴D electrode. From left to right: C1: front view of the PCB design drawing; C2: back view of the PCB design drawing and C3: frontal view of the physical electrode.

S2. Numerical analysis of MRB-sC^{4}D

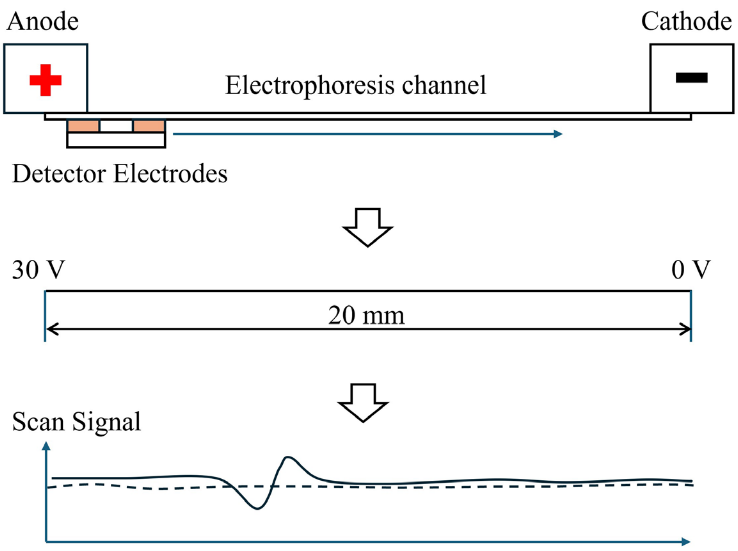

Figure S2. Numerical simulation model of MRB-sC^{4}D

In this work, the detector electrodes slip under the electrophoresis chip to scan the conductivity in the channel. The conductivity in the channel can be calculated by the following equation:

$$\sigma = F(\mu_{\text{eff}}^{+}c^{+} + \mu_{\text{eff}}^{-}c^{-}) \quad \text{(S1)}$$

here, F is the Faraday constant, μ is the ion mobility, c is the ion concentration, the symbols "-" and "+ " denote the cathode and anode respectively.

For ions, the effective mobility μ_i can be calculated using Equation (S2):

$$\mu_i = \frac{ez}{6\pi\eta r} \quad \text{(S2)}$$

The above equation means the conductivity in the channel was determined by the species and concentration of ions that can be driven by an electric field. In the electrophoresis system, the system was electric neutrality.

$$\sum_i z_i^{+} c_i^{+} + \sum_i z_i^{-} c_i^{-} = 0 \quad \text{(S3)}$$

The ions migration could be described by the convection diffusion equation:

$$\frac{\partial c_i}{\partial t} + \nabla(-\mu_i c_i E + D_i \nabla c_i) = R_i \quad \text{(S4)}$$

where, ci is the concentration of species i, t means time, μ is the effective mobility of species i, E is the electric field, Di is the diffusion coefficient and Ri means the reaction rate which is related to the ET reaction.

In the simulation model, the total length of channel is 20 mm. Since the channels are filled with gel, both fluid convection and electroosmotic flow effects become negligible during electrophoresis. The anode potential is set at 30V, while the cathode potential is fixed at 0V.

The parameters used in the simulation model were listed in the Table S1. Initial concentrations in the channel were given as following:

The concentrations of NaAc and KCl were both set to 1 μM. The boundary conditions at the two ends of the channel were defined as follows: the ion concentration at the cathode end was fixed at the specified value, while the anode end was set to zero. It should be noted that, for CV^{+}

ions, the boundary condition at the anode end was defined as a constant flux, with the specific flux value estimated from experimental measurements.

The ion concentration and conductivity distributions within the channel, calculated for CV^+ concentration of 2 μM at an electrophoresis time of 11 minutes, were shown in Figure S3. The conductivity distributions at different time points were presented in Figure S4.

Table S1 Diffusion coefficients and electrophoretic mobilities of ions in the numerical simulation model.

Species	Diffusion coefficient (m^2/s)	Electrical mobility ($m^2/V/s$)
H^+	9.31e-9	3.62e-7
OH^-	5.27e-9	2.05e-7
Na^+	5.28e-9	5.19e-8
K^+	1.96e-9	7.62e-8
Cl^-	2.03e-9	7.91e-8
Ac^-	1.09e-9	4.24e-8
CV^+	3.50e-10	3.90e-9

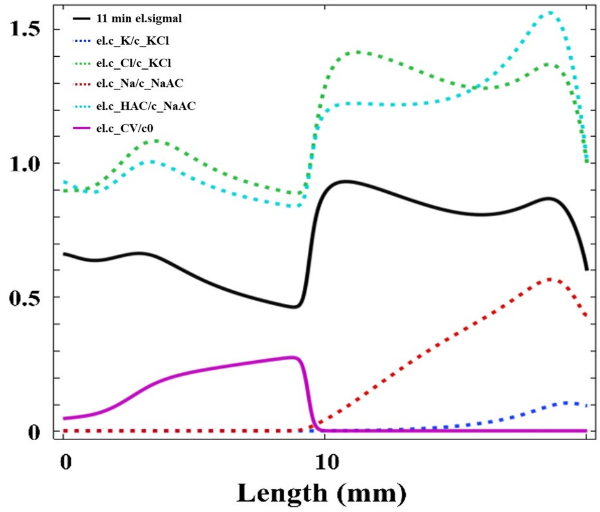

Figure S3 Numerical simulation results of ion concentration and conductivity distributions in the electrophoresis channel at an electrophoresis time of 11 minutes.

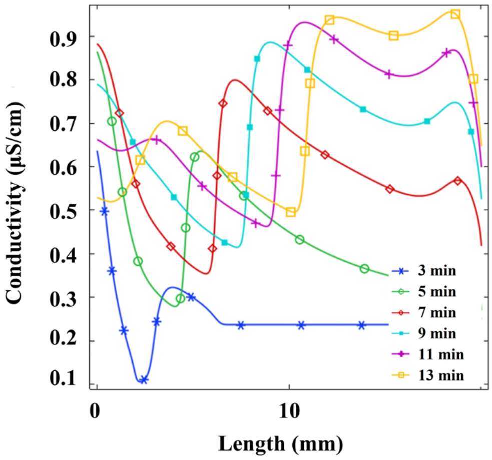

Figure S4. Simulation results of conductivity distributions in the channel at different time.

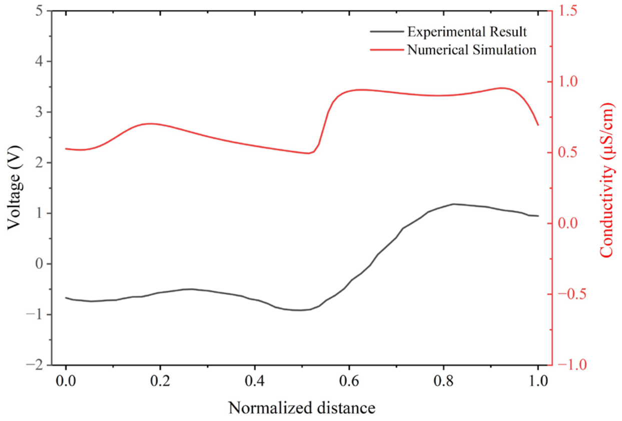

Figure S5 Overlay of the simulated and experimental conductivity profiles.

S3. The Experiment of Optical ET

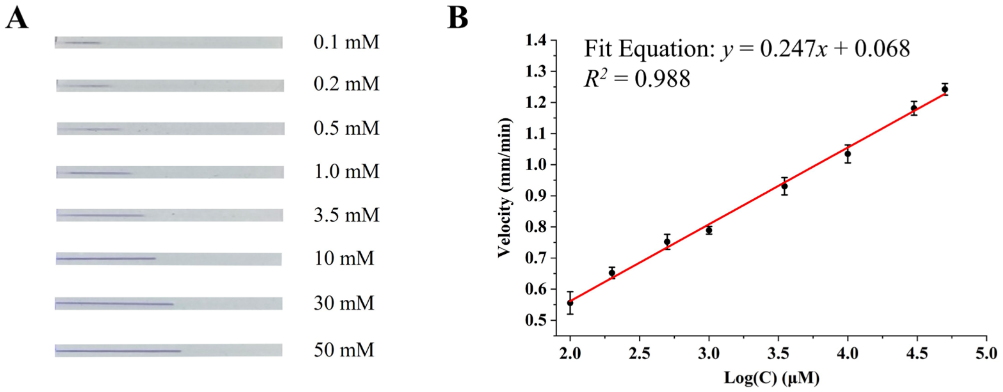

Figure S6. The experimental results of optical ET for Glu detection. (A). Photo of 10 min boundary movement under different Glu concentration. (B). The linear fitting curve between the velocity of boundary movement and the logarithm of Glu concentration.

S4. The Quantification Detection of Creatinine and Choline

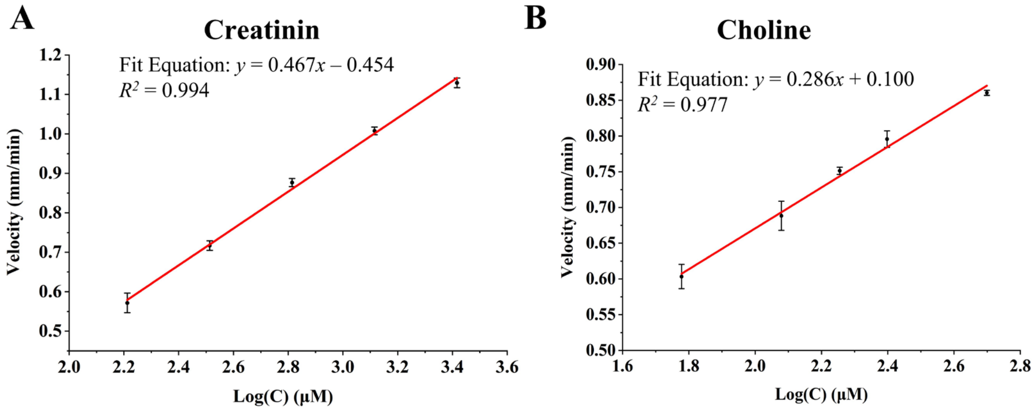

Figure S7. The quantification detection of creatinine and choline. (A). The linear fitting curve between the velocity of boundary movement and the logarithm of creatinine concentration (n=3), with linear range: 0.16-2.6 mM. (B). The linear fitting curve between the velocity of boundary movement and the logarithm of choline concentration (n=3), with linear range: 60-500 μM.
